# Immunological response and temporal associations in myocarditis after COVID-19 vaccination using cardiac magnetic resonance imaging: An amplified T-cell response at the heart of it?

**DOI:** 10.3389/fcvm.2022.961031

**Published:** 2022-09-15

**Authors:** Hajnalka Vago, Liliana Szabo, Zsofia Szabo, Zsuzsanna Ulakcsai, Emese Szogi, Gizella Budai, Attila Toth, Vencel Juhasz, Zsofia Dohy, Krisztina Hoffer, David Becker, Robert Gabor Kiss, Gergely Gyorgy Nagy, Gyorgy Nagy, Bela Merkely

**Affiliations:** ^1^Heart and Vascular Center, Semmelweis University, Budapest, Hungary; ^2^Department of Sports Medicine, Semmelweis University, Budapest, Hungary; ^3^Queen Mary University of London, London, United Kingdom; ^4^Department of Laboratory Medicine, Semmelweis University, Budapest, Hungary; ^5^Department of Anesthesiology and Intensive Therapy, Semmelweis University, Budapest, Hungary; ^6^Military Hospital, Budapest, Hungary; ^7^Borsod-Abaúj-Zemplén County Central Hospital and University Teaching Hospital, University of Debrecen, Debrecen, Hungary; ^8^Department of Radiology, Erzsébet Teaching Hospital and Rehabilitation Institute of Sopron, Sopron, Hungary; ^9^Department of Rheumatology and Clinical Immunology, Semmelweis University, Budapest, Hungary; ^10^Department of Genetics, Cell- and Immunobiology, Semmelweis University, Budapest, Hungary

**Keywords:** myocarditis, SARS-CoV-2 immunization, cardiovascular magnetic resonance, immunological response, vaccination, inflammation

## Abstract

**Introduction:**

Although myocarditis after anti-SARS-CoV-2 vaccination is increasingly recognized, we have little data regarding the course of the disease and, consequently, the imaging findings, including the tissue-specific features. The purpose of this study is to describe the clinical, immunological, and cardiac magnetic resonance (CMR) features of myocarditis after COVID-19 immunization in the acute phase and during follow-up. We aimed to compare the trajectory of the disease to myocarditis cases unrelated to COVID-19.

**Methods:**

We assembled a CMR-based registry of potentially COVID-19 vaccination-related myocarditis cases. All patients who experienced new-onset chest pain and troponin elevation after COVID-19 vaccination and imaging confirming the clinical suspicion of acute myocarditis were enrolled in our study. Participants underwent routine laboratory testing and testing of their humoral and cellular immune response to COVID-19 vaccination. Clinical and CMR follow-up was performed after 3–6 months. We included two separate, sex- and age-matched control groups: (1) individuals with myocarditis unrelated to COVID-19 infection or vaccination confirmed by CMR and (2) volunteers with similar immunological exposure to SARS-CoV-2 compared to our group of interest (no difference in the number of doses, types and the time since anti-SARS-CoV-2 vaccination and no difference in anti-nucleocapsid levels).

**Results:**

We report 16 CMR-confirmed cases of myocarditis presenting (mean ± *SD*) 4 ± 2 days after administration of the anti-SARS-CoV-2 vaccine (male patients, 22 ± 7 years), frequently with predisposing factors such as immune-mediated disease and previous myocarditis. We found that 75% received mRNA vaccines, and 25% received vector vaccines. During follow-up, CMR metrics depicting myocardial injury, including oedema and necrosis, decreased or completely disappeared. There was no difference regarding the CMR metrics between myocarditis after immunization and myocarditis unrelated to COVID-19. We found an increased T-cell response among myocarditis patients compared to matched controls (*p* < 0.01), while there was no difference in the humoral immune response.

**Conclusion:**

In our cohort, myocarditis occurred after both mRNA and vector anti-SARS-CoV-2 vaccination, frequently in individuals with predisposing factors. Upon follow-up, the myocardial injury had healed. Notably, an amplified cellular immune response was found in acute myocarditis cases occurring 4 days after COVID-19 vaccination.

## Introduction

Increasing evidence links coronavirus disease 2019 (COVID-19) vaccination to rare cases of myocarditis and myopericarditis, primarily in the young adult (1) and adolescent (2) male population (3, 4). The connection between novel mRNA vaccines and these cases has been made. However, earlier data show that post-vaccination myocarditis may occur after a variety of vaccinations, including the smallpox vaccine that contains live virus (5).

Cardiac magnetic resonance (CMR) imaging is the method of choice for noninvasive visualization of myocardial injury (6–8). Case reports and case series demonstrated the role of CMR in the confirmation of myocarditis after anti-SARS-CoV-2 immunization. Importantly, these cases describe vaccine-induced myocarditis associated with mRNA vaccines, particularly after the second dose of the BNT162b2 mRNA-Pfizer-BioNTech and mRNA-1273-Moderna vaccines (9–12). An extensive cohort study from Israel based on hospital reporting systems described clinical follow-up data, but measures of cardiac function were not available (13). Therefore, we have little data regarding the course of the disease and, consequently, the CMR findings, including the tissue-specific features of myocarditis.

The underlying mechanism of the evolution of vaccination-related myocarditis is largely unclear. The proposed concepts include triggering of preexisting immune pathways and accelerated innate immunogenic reactions (4). Previously, it was also suspected that spike reactive mimicry might also play a role; however, this hypothesis has since been refuted by Marram et al. (14). However, these are primarily theoretical notions, as the immune response of myocarditis patients after COVID-19 vaccination has not been described (4).

The purpose of this study was to describe the clinical, CMR imaging and immunological features of different types of myocarditis after COVID-19 immunization in the acute phase and during follow-up. Second, we aimed to illustrate the features of myocarditis potentially linked to the COVID-19 vaccine in the context of myocarditis cases where vaccination or any contact with COVID-19 disease did not occur. Third, we describe the immunological response to COVID-19 immunization in patients with myocarditis and matched controls.

## Methods

### Study population

This is a retrospective CMR-based registry of myocarditis cases following COVID-19 immunization. We contacted all Hungarian institutions performing CMR scans (*n* = 19) between December 2020 and September 2021. All participants must exhibit the following inclusion criteria, to be admitted to the study: (1) COVID-19 vaccination not more than 21 days before the acute presentation; (2) presence of one or more of the following symptoms: new-onset chest pain, dyspnea, or palpitation or syncope; (3) troponin elevation as per the local laboratory; and (4) CMR imaging confirming the clinical suspicion of acute myocarditis. Based on our criteria, four centers reported myocarditis cases after COVID-19 vaccination.

### Study protocol

**Figure 1:**
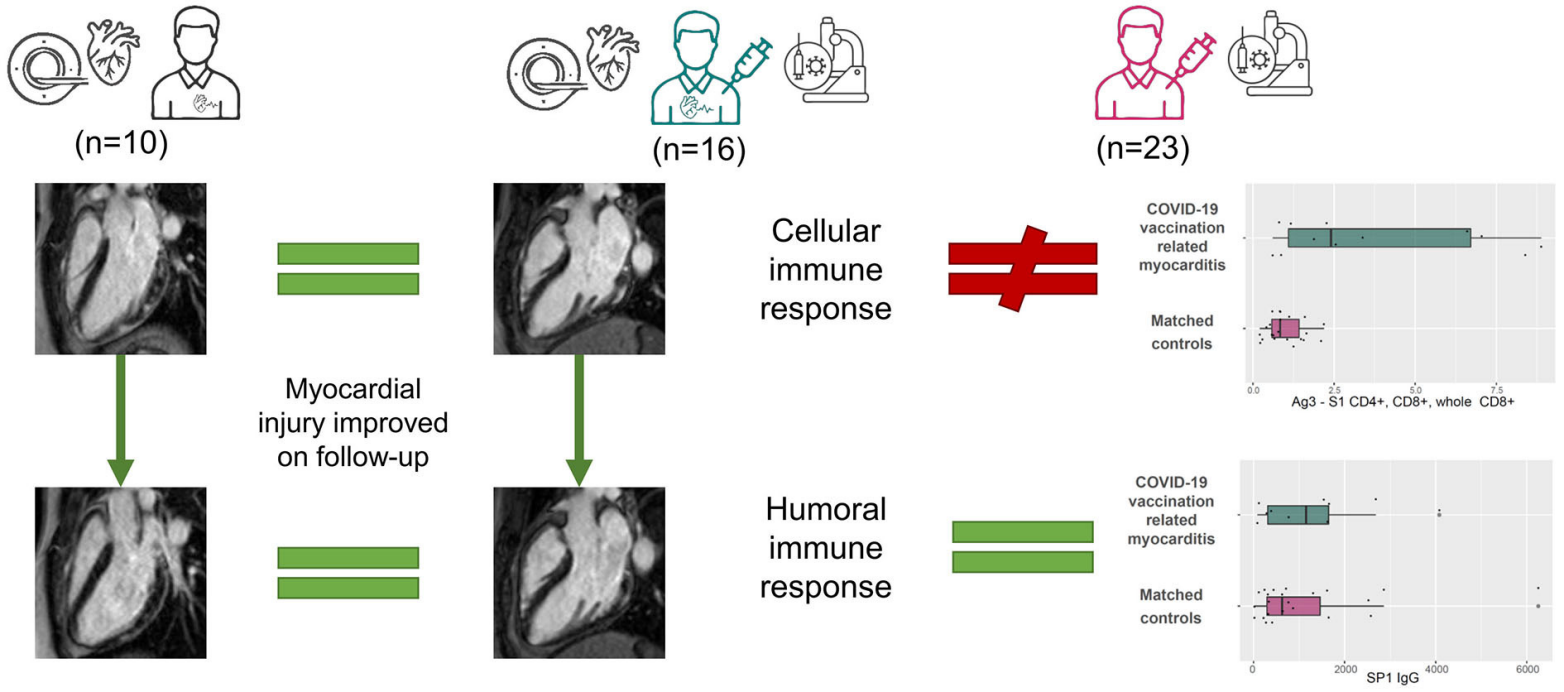
Visual abstract. We compared the CMR findings of myocarditis patients after COVID-19 vaccination (middle) to those of patients with myocarditis unrelated to COVID-19 immunization or infection (left). We did not find a difference between the groups in the acute (upper images) or follow-up (lower images) scans, but the myocardial injury improved. We compared the immune response of myocarditis patients after COVID-19 vaccination to COVID-19 immunization status-matched controls (right). There was no difference regarding the humoral immune response. In contrast, the cellular immune response was amplified in the myocarditis group.

All participants completed a questionnaire regarding their acute symptoms and previous medical history, including their history of cardiovascular and immunological diseases. Cardiac biomarker levels, laboratory test results and 12-lead ECG results were recorded. Echocardiography and CMR examination were performed. Immunological tests were carried out in all acquiescent participants. Symptomatic patients (e.g., ongoing chest pain) were admitted to intensive/coronary care units (ICU/CCU) with continuous bedside monitoring. Asymptomatic patients with elevated cardiac troponin or patients discharged from ICU/CCU to general wards were monitored using telemetry. Follow-up examinations and CMR scans were carried out 3–6 months after the acute presentation in patients who consented. The study design is shown on Figure 1.

### Ethical approval

Ethical approval was obtained from the National Public Health Center under the ethical standards laid out in the 1964 Declaration of Helsinki and its later amendments. IV/2568-1/2021/EKU. All participants or their legal guardian gave their written informed consent for the analysis.

### Myocarditis comparator group

We included a group of myocarditis patients confirmed by CMR to illustrate the potential similarities and differences from the myocarditis patients after COVID-19 vaccination. The CMR comparator group was sex- and age-matched, retrospectively selected from the Semmelweis University CMR database according to the following criteria: (1) troponin elevation, (2) CMR examination confirming acute myocarditis was completed <2 weeks after the acute presentation, (3) CMR examination before the first reported case of SARS-CoV-2 infection in Hungary (2020.03.04.) OR negative PCR excluding the infection, and (4) follow-up CMR was carried out between 3 and 6 months after the acute scan. All control CMR scans were performed using a Siemens Magnetom Aera 1.5 T scanner. A comprehensive CMR protocol was carried out, including cine movies, T2-weighted spectral presaturation with inversion recovery (SPIR), T2 mapping using T2-prep balanced steady-state free precession (b-SSFP), T1 mapping using long-T1 5(3)3 and short-T1 5(3)3 modified look-locker inversion recovery (MOLLI) and late gadolinium enhancement (LGE) imaging. Functional evaluation was performed using b-SSFP cine sequences in four-chamber, two-chamber, and three-chamber long-axis views and a short-axis stack from the cardiac base to apex with full coverage of the left ventricle and right ventricle. None of the myocarditis patients had a history of immune checkpoint inhibitor treatment.

### CMR protocol

Overall, four Hungarian centers reported myocarditis cases after SARS-CoV-2 vaccination. CMR scans were performed on 1.5 T scanners (Siemens Magnetom Aera, Siemens Magnetom Amira, GE SIGNA Voyager, Phillips Ingenia). The CMR protocol had to include the following sequences regardless of the institution: cine sequence covering the whole heart for functional assessment, T2 weighted images or T1 mapping depicting myocardial oedema and LGE or T1 mapping showing necrosis or fibrosis. The protocol of the acute and control CMR scans was similar in most cases, although we accepted control CMR scans without T2-weighted images. If a control CMR scan was not possible in the original institution, the participant was offered a CMR scan slot at the Semmelweis University Heart and Vascular Center (*n* = 2). Mapping sequences were available from 3 institutions (*n* = 13/16). LGE images were acquired using segmented inversion recovery sequences 10–15 min after administration of an intravenous bolus of gadolinium-based contrast agent (gadobutrol in 0.15 ml/kg, or gadoteric acid in 0.4 ml/kg) at a rate of 2–3 ml/s through an antecubital intravenous line. The inversion time was adjusted to provide optimal suppression of normal myocardium.

### CMR analysis

CMR scans were collected in raw DICOM format, and all post-processing analyses were conducted in a core CMR laboratory using the Medis Suite Software (Medis Medical Imaging Software, The Netherlands) to minimize observer-related variance. LV and RV volumes, function and mass were calculated from the SA stack using artificial intelligence-based automated contour detection (autoQ module) with manual adjustments if necessary. Short-axis LGE images were contoured manually, and then the LGE mass and LGE% were quantified using the 5SD technique with manual adjustments if required in the Medis QMass module. Myocardial native T1 and T2 relaxation times were consequently measured in the midventricular or basal septum (15) (if the midventricular images were technically inadequate for analysis) of the myocardium using motion-corrected images. One further ROI was manually drawn to the affected area guided by visual inspection (15). The comparison regarding mapping values was carried out in participants who underwent their CMR examination and at Semmelweis University Heart and Vascular Center (*n* = 9). Elevated T1 and T2 values were defined based on sequence-specific cut-offs of 2 standard deviations (SDs) above the respective means of the healthy male controls (T1: 1,000 ms, T2: 49 ms).

Acute myocarditis was defined as per the modified Lake Louise criteria (LLC) (7). Specifically, at least two positive main LLC criteria in corresponding locations were necessary for the diagnosis. At least one positive criteria for oedema visualization (T2-weighted images, T2 mapping or T1 mapping) and at least one positive criteria for necrosis visualization (LGE or T1 mapping). The interpretation of CMR scans was standardized: the presence and pattern of myocardial oedema and LGE was visually defined independently by two EACVI certified observers (VH EACVI level 3-certified CMR specialists with more than 15 years of experience in CMR reporting and LS completed her EACVI written certification and has 3.5 years of experience reporting CMR). In case of disagreement between the observers, a third level 3 EACVI-certified CMR specialist (AT) with more than 15 years of experience in CMR reporting was consulted for consensus. Non-ischaemic LGE was defined as midmyocardial and/or subepicardial myocardial LGE confirmed in two perpendicular views.

### Control group for immunological studies

The immune response of the study participants was compared with that of 23 sex- and age-matched controls from the Semmelweis University database. Subjects included in the control group were comparable to the myocarditis group regarding the doses and type of anti-SARS-CoV-2 vaccine they received and the time elapsed since their vaccination. We objectively quantified SARS-CoV-2 exposure using anti-nucleocapsid protein levels, which showed no difference between myocarditis patients after COVID-19 vaccination and controls. This matching step was crucial, as more participants reported previous SARS-CoV-2 infection in the control group than in the myocarditis group.

### Laboratory protocol

Participants underwent routine laboratory testing for biomarkers including troponin, CKMB, CRP, white blood cell count, and eosinophil cell count. Antinuclear antibodies (ANAs), extractable nuclear antigen antibodies (ENAs), antineutrophil cytoplasmic antibodies (ANCAs) and serum immunoglobulin (IgG, IgM, IgA) levels were also measured from myocarditis samples (*n* = 10). A subgroup of myocarditis patients after COVID-19 vaccination (*n* = 12) and all immunization-matched controls (*n* = 23) underwent an evaluation of humoral and cellular immune responses at Semmelweis University. The immunology protocol and their interpretation were standardized to allow meaningful comparisons. Enzyme immunoassay providing semiquantitative *in vitro* determination of human antibodies of the immunoglobulin class IgG and IgM against modified nucleocapsid protein (NCP) of SARS-CoV-2 in serum or plasma has been obtained (referred to in the text as NCP-IgG and NCP-IgM). The results are given as a ratio (extinction of the sample/extinction of calibrator). The results below 0.8 are considered negative, the results equal to or above 0.8 and below 1.1 are considered borderline, and the results equal to or above 1.1 are considered positive due to the test description. SARS-CoV-2-specific antibodies (referred to in the text as S1 Ig) were analyzed using an Elecsys Anti-SARS-CoV-2 S immunoassay (Roche Diagnostics International Ltd, Switzerland) on a Cobas e6000 machine. The test detects antibodies specific to the SARS-CoV-2 spike (S) protein receptor-binding domain (RBD) in human serum and plasma. The method uses electrochemiluminescence to quantitatively determine antibodies based on the double-antigen sandwich principle. The test cut-off was ≥0.8 as per the manufacturer. The detailed immunoglobulin response was determined using the ELISA test, and the sample dilution was performed manually; further steps were carried out automatically using an Elite Lite (DAS, Italy) device. We will refer to the IgG and IgA immunoglobulins recognizing the S1 domain of the spike protein determined by ELISA as SP1 IgG and IgA for transparency. We quantified immunoglobulin levels in a quantitative (SP1 IgG) or semiquantitative (SP1 IgA) manner (16). The T-cell response was assessed via the QuantiFERON SARS-CoV-2 assay, an interferon-gamma release assay described in detail elsewhere (17). In short, this assay consists of three antigen tubes, SARS-CoV-2 Ag1, Ag2 and Ag3, that use a combination of proprietary antigen peptides specific to SARS-CoV-2 to stimulate lymphocytes involved in cell-mediated immunity in heparinized whole blood. The Ag1 tube contains CD4+ epitopes derived from the S1 subunit RBD of the spike protein. The Ag2 tube contains CD4+ and CD8+ epitopes from the S1 and S2 subunits of the spike protein. The Ag3 tube consists of CD4+ and CD8+ epitopes from S1 and S2 and immunodominant CD8+ epitopes derived from the whole SARS-CoV-2 genome.

### Data management and statistical analysis

Statistical analysis and data visualization were performed using MedCalc software V.18.11 (Belgium) and RStudio (Version 1.3.1.093, RFoundation, Austria). The Shapiro–Wilk test was applied to test the normality of our data. Continuous variables showing a normal distribution are presented as the mean and SD, and those showing a non-normal distribution are reported as medians and IQRs. Categorical variables are presented as frequencies and percentages. Acute and follow-up examinations were compared using paired sample *t* tests and Wilcoxon tests. We applied analysis of covariance (ANCOVA) to formally test the difference between the trajectory of myocarditis after SARS-CoV-2 vaccination and myocarditis unrelated to COVID-19. Chi tests were applied to compare the distributions of categorical data. Comparisons between the immunological response of myocarditis patients after SARS-CoV-2 vaccine and the comparator group were conducted using independent samples *t* tests and Mann–Whitney *U* tests as appropriate. Associations were assessed using Spearman's rank correlation analyses. Probability values were two-sided, and *p* values of < 0.05 were considered significant. All data are available on reasonable request.

## Results

### Description of clinical characteristics

**Figure 2:**
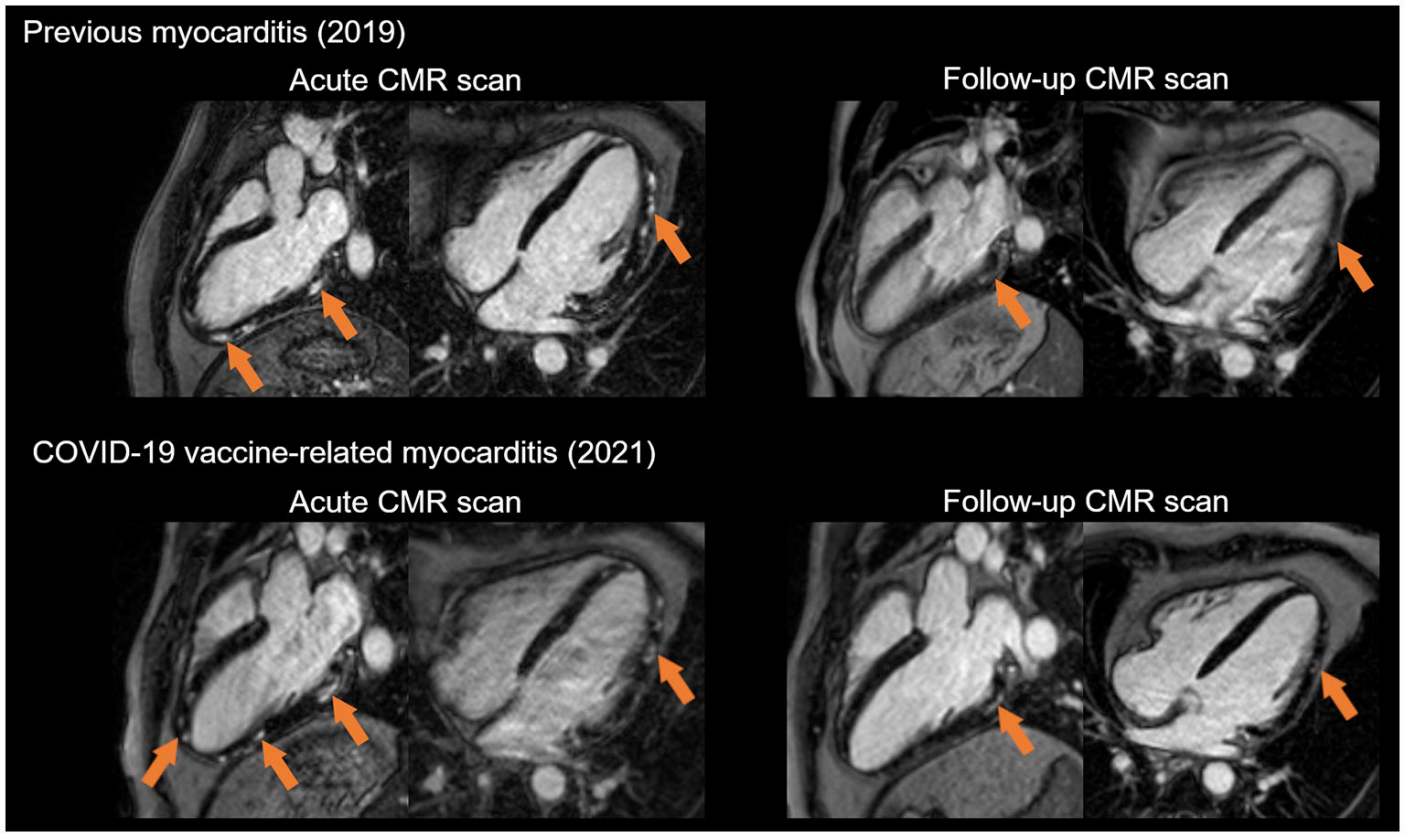
Recurrent myocarditis in a young male patient after the second dose of anti-COVID-19 vector vaccine. Our patient had prior myocarditis in 2019. At the time, he presented with chest pain preceded by gastrointestinal infection and fever. He had elevated troponin levels, and the CT coronary angiogram was negative. The acute CMR showed patchy subepicardial oedema and late gadolinium enhancement (LGE) (orange arrows). Three months later, on his follow-up scan, the oedema disappeared, and the LGE shrank. In 2021, the patient experienced fever and recurrent chest pain 2 days after the second dose of the COVID-19 vaccine. His acute CMR imaging showed LGE in a similar pattern as during the first acute myocarditis episode. Notably, signs of myocardial injury resolved on the follow-up scan.

A total of four centers reported 16 CMR-confirmed cases of myocarditis following SARS-CoV-2 immunization, with chest pain presenting a mean of 4 ± 2 days after vaccination. Patient characteristics are included in Table 1. All of them were young (five were <18 years, mean age 22 ± 7 years, between 13 and 36 years) male patients and generally presented after their second dose of COVID-19 immunization (13, 81%). Most of them received mRNA vaccines (75%), while 25% presented with myocarditis after receiving a vector vaccine. Three patients reported prior SARS-CoV-2 infection, and one of them developed acute myocarditis after the first dose of vaccine. Two participants had acute myocarditis in their previous medical history confirmed by CMR imaging (Figure 2). In these cases, the time elapsed from the prior myocarditis to vaccination was 2 and 4 years, respectively. Four patients reported immune-mediated diseases, including Crohn's disease, psoriasis, asthma and allergies. None of the patients received systemic corticosteroid therapy. Overall, four participants reported intensive physical activity directly after vaccination (intensive sport activity, heavy physical labor), and one individual noted heavy alcohol consumption following immunization. The first systemic symptoms (fever, shivering) developed within 2 days, and chest pain presented a mean of 4 days after vaccination in all patients. ECG alterations were documented in seven patients (ST elevation in 6, negative T wave in 1). The initial troponin level was elevated in all study participants (Table 2), and we frequently noted CKMB, CRP and proBNP elevation as well. The white blood cell count, eosinophil count, and other markers remained in the normal range. During the acute phase, there were no heart failure symptoms, syncope, or documented sustained brady- or tachyarrhythmias.

**Table 1:** Baseline characteristics.

Age, years	22 ± 7
Sex, male %	16 (100)
BMI	26 ± 4
**SARS-CoV-2 vaccine type** ***n*****, (%)**	
mRNA	
- Pfizer (BNT162b2 mRNA-Pfizer- BioNTech)	10 (62.5)
- Moderna (mRNA-1273-Moderna)	2 (12.5)
Vector vaccine	
- Sputnik V (Gam-COVID-Vac)	4 (25)
SARS-CoV-2 vaccine dose ***n***, (%)	
- First dose	2 (12.5)
- Second dose	13 (81.2)
- Third dose	1 (6.2)
First complaint after vaccination, days	1.8 ± 1.6
Chest pain after vaccination, days	3.8 ± 1.9
Previous SARS-CoV-2 infection yes, *n* %	2 (12.5)
Previous myocarditis yes, *n* %	2 (12.5)
Positive immunological history	4 (25)
- Crohn's disease, *n* %	1 (6.2)
- Asthma, *n* %	1 (6.2)
- Psoriasis, *n* %	1 (6.2)
- Allergy, *n* %	1 (6.2)
Cardiovascular risk factors	
- Hypertension, *n* %	2 (12.5)
- Diabetes, *n* %	0 (0)
- Smoking, *n* %	4 (25)
- Obesity, *n* %	3 (18.8)
Intense physical activity after vaccination	4 (25)
- Sport activity	3 (18.8)
- Physically demanding job	1 (6.2)
Elevated troponin level *n*, %	16 (100)
CKMB (U/L) *Cut-off: ≥ 25 U/L*	31 [26, 62]
C-reactive protein (mg/L) *Cut-off: ≥ 5 mg/L*	23 [13, 43]
NTproBNP (pg/ml) *Cut-off: ≥ 125 pg/ml*	351 [223, 677]
Thrombocyte count (Giga/L) *Normal range: 150–400 Giga/L*	214 [199, 229]
White blood cell count (Giga/L) *Normal range: 4.0–10.0 Giga/L*	7.9 [5.7, 9.5]
Eosinophil count (Giga/L) *Cut-off: >0.5 Giga/L*	0.10 [0.07, 0.17]

Baseline characteristics.

CKMB, Creatine kinase-MB; NTproBNP, N-terminal pro B-type natriuretic peptide; SARS-CoV-2, Severe acute respiratory syndrome coronavirus 2.

**Table 2:** Peak troponin value for myocarditis patients after COVID-19 vaccination.

**Case no**	**Cardiac troponin type**	**Local cut-off**	**Peak value**
1	hs troponin T (ng/L)	>14 ng/L	1,159
2	hs troponin T (ng/L)	>14 ng/L	1,007
3	hs troponin T (ng/L)	>14 ng/L	376
4	hs troponin T (ng/L)	>14 ng/L	1,366
5	hs troponin T (ng/L)	>14 ng/L	3,018
6	hs troponin T (ng/L)	>14 ng/L	144
7	hs troponin I (pg/ml)	>19 gp/ml	11,907
8	hs troponin I (μg/L)	>0.0198 μg/L	4.067
9	hs troponin T (ng/L)	>14 ng/L	2,136
10	hs troponin T (ng/L)	>14 ng/L	212
11	hs Troponin I (pg/ml)	>34.2 pg/ml	7,665
12	hs troponin T (ng/L)	>14 ng/L	220
13	hs troponin T (ng/L)	>14 ng/L	2,431
14	Troponin I (ng/L)	>19 ng/L	4,047
15	hs troponin I (pg/L)	>30 gp/ml	3,976
16	hs troponin T (ng/L)	>14 ng/L	228

Maximal troponin values for each participants is reported according to the local laboratory. hs, high-sensitive.

### CMR features of acute myocarditis after COVID-19 immunization

**Figure 3:**
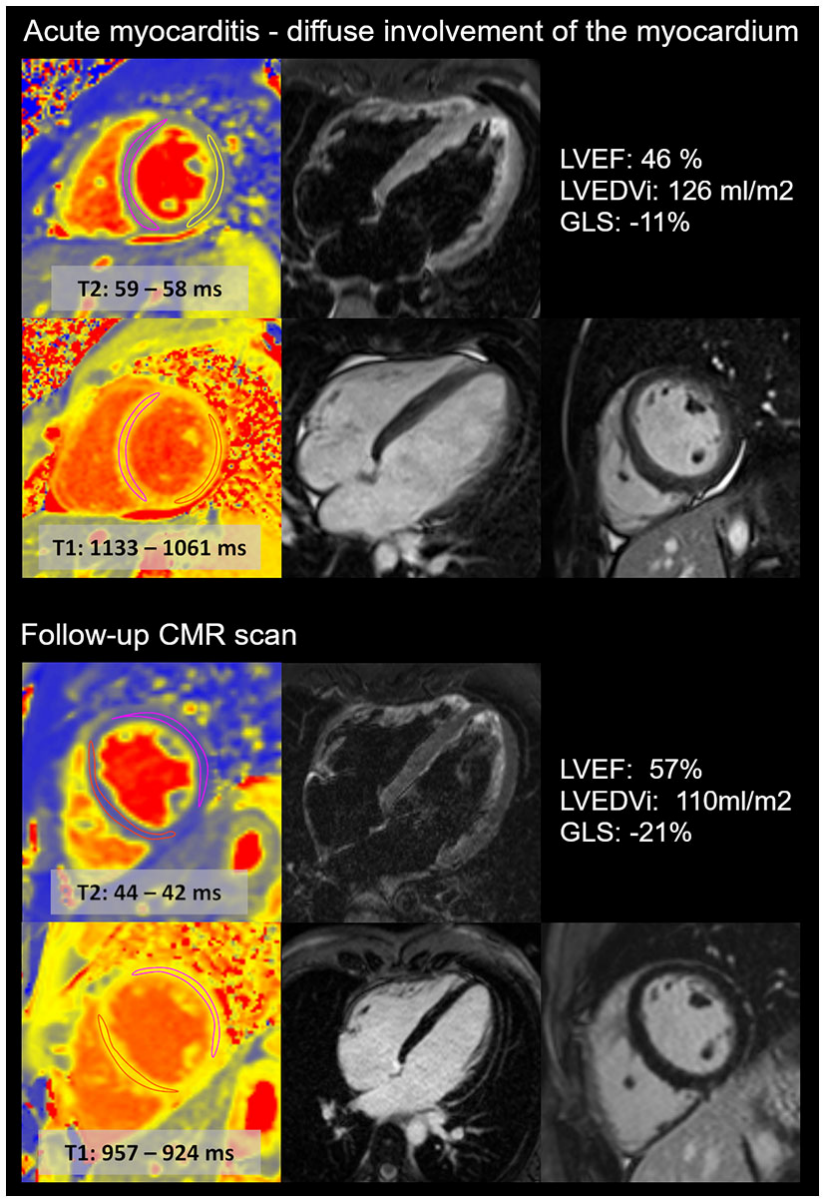
Diffuse acute myocarditis after the second dose of anti-COVID-19 mRNA vaccine in a young athlete. CMR images show the acute (upper images) and follow-up (lower images) scans of a young, highly trained athlete (national team member). The first CMR scan confirmed acute myocarditis with diffuse involvement of the myocardium, with elevated T2 and T1 mapping and diffuse myocardial oedema. The left ventricular ejection fraction (LVEF) was mildly decreased, and global longitudinal (GLS) strain was decreased during the acute scan. The follow-up scan revealed the normalization of T2 and T1 mapping values and left ventricular systolic function. The LVEDVi decreased. No LGE was present. The patient was prohibited from participation in sports activity for the first 3 months, and then he gradually returned to sports activity. Currently, the athlete performs a high level of sports activity and does not report recurrent or persisting symptoms.

CMR was performed on average 4 ± 2 days (between 1 and 8 days) after the onset of acute chest pain. The majority of the cases showed a localized pattern of myocarditis, mainly affecting the lateral wall of the left ventricle with signs of subepi-midmyocardial oedema and necrosis (Figure 2). In one case, we found diffuse myocarditis with elevated T2, T1 and ECV values (Figure 3) caused by the mRNA vaccine. The left ventricular ejection fraction (LVEF) was in the normal range for most cases, except for two patients whose LVEF was mildly decreased (46 and 47%). Notably, these two patients had no previous history of acute myocarditis. There was no definitive pericardial involvement in any patients.

### Clinical status and CMR changes during follow-up

During our follow-up, one patient experienced a recurrent episode of acute myocarditis (3 months after the vaccine), preceded by gastrointestinal infection. Other patients did not report symptom recurrence. The hs Troponin T (6[4, 7] ng/L), CKMB (2[2, 11] U/L), CRP (2[1, 3] mg/L) and proBNP (29[12,49] pg/ml) values returned to the normal range. Follow-up CMR was carried out 112 ± 27 days after the baseline scan (*n* = 14). We found that the LVEF marginally increased upon follow-up, and LVEDVi slightly decreased, both remaining in the normal range (Table 3). Elevated T2 values depicting local oedema in the affected area were resolved. The native T1 value and ECV measured in the affected area also decreased; however, ECV remained slightly elevated. The LGE area shrank in all participants and disappeared completely in 31% (4/13) of cases. The highly trained athlete in whom all signs of acute myocarditis disappeared on follow-up (Figure 3) was able to gradually return to sports activity. He restarted exercising 3 months ago and did not experience recurrent or persisting symptoms.

**Table 3:** Comparison between acute and follow-up CMR scans of myocarditis patients after COVID-19 vaccination.

	**Acute myocarditis after COVID-19 vaccination** **(*n* = 16)**	**Follow-up myocarditis after COVID-19 vaccination** **(*n* = 14)**	**Acute *vs*. follow-up CMR, myocarditis after COVID-19 vaccination** **(*P* values)**
Elapsed time, days	4 ± 2	112 ± 27	NA
LVEF, %	58 ± 6	60 ± 3	0.042
LVEDVi, ml/m^2^	87 ±13	83 ± 9	0.046
LVSVi, ml/m^2^	50 ± 7	50 ± 6	0.961
LVMi, g	53 ± 10	51 ± 7	0.228
GLS, %	−20.5 [−22.5, −19]	−21 [−22, −20]	0.083
RVEF, %	58 ± 4	57 ± 5	0.559
RVEDVi, ml/m^2^	83 ± 10	84 ± 9	0.722
RVSVi, ml/m^2^	48 ± 6	48 ± 6	0.489
T1 mapping septal, ms	966 [951, 1,016]	957 [950, 965]	0.578
T1 mapping affected area, ms	1,056 [1,038, 1,113]	976 [953.5, 1,018]	0.031
T2 mapping septal, ms	43 [43, 44]	43 [42, 43]	0.375
T2 mapping affected area, ms	51 [50, 55]	44 [43, 47.5]	0.016
ECV septal, %	26 [24, 28]	25.5 [23.5, 27.5]	0.125
ECV affected area,%	38 [35, 41.5]	30.5 [28, 35]	0.016
LGE g	6 [3, 10]	2 [0.5, 4]	0.001
LGE %	7 [3, 12]	3 [1, 4]	0.001

Comparison between acute and follow-up CMR scans myocarditis after COVID-19 immunization. Continuous variables showing a normal distribution are presented as the mean and standard deviations (± SD), and those showing a non-normal distribution are reported as medians and interquartile ranges [IQRs]. Acute and follow-up examinations were compared using paired sample t tests and Wilcoxon tests.

CMR, cardiac magnetic resonance; ECV, extracellular volume; EDVi, left ventricular end diastolic volume index; EF, ejection fraction; ESVi, end systolic volume index; GLS, global longitudinal strain; Mi, mass index; NA, not applicable; LGE, late gadolinium enhancement; LV, left ventricular; RV, right ventricular; SVi, left ventricular stroke volume index.

### Myocarditis after SARS-CoV-2 immunization vs. myocarditis unrelated to COVID-19

**Figure 4:**
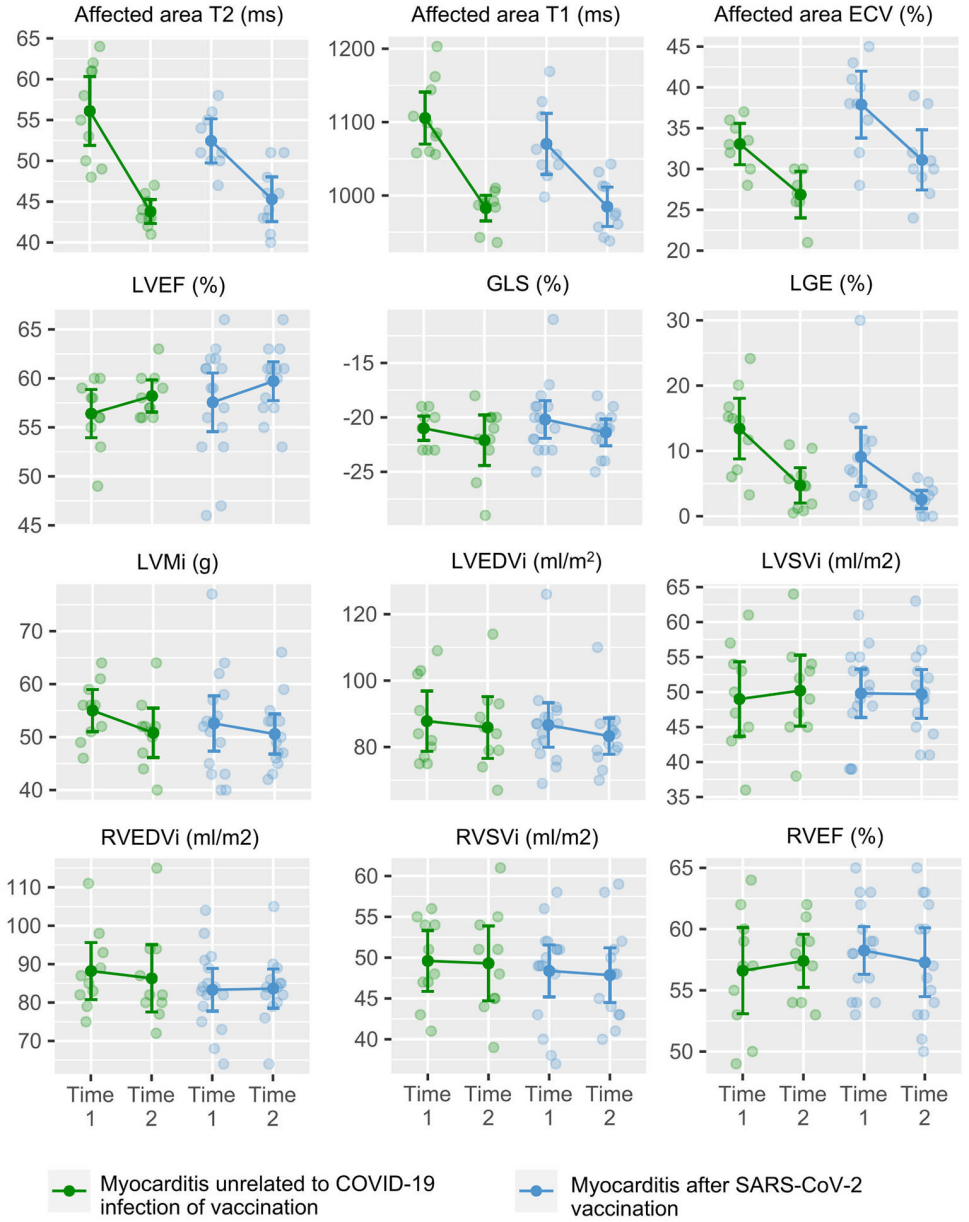
CMR metrics of myocarditis patients after SARS-CoV-2 immunization and myocarditis patients unrelated to COVID-19 immunization or infection over the acute phase and follow-up scan. Graphs show the trajectory of CMR metrics between the acute (T1) and follow-up (T2) CMR scans in myocarditis patients after SARS-CoV-2 immunization (in blue) and myocarditis patients unrelated to COVID-19 infection or vaccination (in green). CMR, cardiac magnetic resonance; ECV, extracellular volume; EDVi, left ventricular end diastolic volume index; EF, ejection fraction; ESVi, end systolic volume index; GLS, global longitudinal strain; Mi, mass index; LGE, late gadolinium enhancement; LV, left ventricular; RV, right ventricular; SVi, left ventricular stroke volume index.

The considering the effect of both follow-up time and myocarditis group, the ANCOVA test showed no difference between the trajectory of cardiac volumes, function, mass, oedema and LGE between myocarditis patients immunization and age- and sex-matched myocarditis patients unrelated to COVID-19 vaccination or infection (male patients, 22 ± 7 vs. 23 ± 6 years). Notably, we found a marginal difference between T1 mapping (Table 4). Figure 4 illustrates the trajectory of CMR metrics between acute and follow-up scans in the both groups.

**Table 4:** Assessment of the trajectory of myocarditis patients after SARS-CoV-2 immunization and myocarditis patients unrelated to COVID-19 immunization or infection over the acute phase and follow-up using analysis of covariance.

**CMR metricss**	**Effects**	**ANCOVA test**
		***P***
LVEF, %	Group	0.476
	Group:Time	0.613
	Time	0.013
LVEDVi, ml/m2	Group	0.752
	Group:Time	0.445
	Time	0.044
LVSVi, ml/m2	Group	0.954
	Group:Time	0.599
	Time	0.641
LVMi, g	Group	0.676
	Group:Time	0.548
	Time	0.051
GLS, %	Group	0.318
	Group:Time	0.812
	Time	0.102
RVEF, %	Group	0.701
	Group:Time	0.384
	Time	0.924
RVEDVi, ml/m2	Group	0.435
	Group:Time	0.501
	Time	0.253
RVSVi, ml/m2	Group	0.601
	Group:Time	0.795
	Time	0.527
T1 mapping septal	Group	0.171
	Group:Time	0.382
	Time	0.002
T1 mapping affected area	Group	0.513
	Group:Time	0.04
	Time	<0.001
T2 mapping septal	Group	0.278
	Group:Time	0.741
	Time	0.075
T2 mapping affected area	Group	0.467
	Group:Time	0.175
	Time	<0.001
ECV septal	Group	0.041
	Group:Time	0.852
	Time	0.112
ECV affected area	Group	0.035
	Group:Time	0.92
	Time	<0.001
LGE g	Group	0.32
	Group:Time	0.554
	Time	<0.001
LGE %	Group	0.164
	Group:Time	0.438
	Time	<0.001

Analysis of covariance (ANCOVA) test results are shown for each CMR metrics, taking into account the effect of the patient group (myocarditis patients after SARS-CoV-2 vaccination vs. myocarditis not linked to SARS-CoV-2 infection) and time of the CMR scan (acute vs. follow-up CMR scan) and the combination of these effects. Models are unadjusted.

CMR, cardiac magnetic resonance; ECV, extracellular volume; EDVi, left ventricular end diastolic volume index; EF, ejection fraction; ESVi, end systolic volume index; GLS, global longitudinal strain; Mi, mass index; LGE, late gadolinium enhancement; LV, left ventricular; RV, right ventricular; SVi, left ventricular stroke volume index.

### Assessment of the immunological response

Markers of the SARS-CoV-2 immune response were obtained for 12 patients. The test was performed a mean of 109 and 86 days after the first and second doses, respectively. Similarly, immunological testing was ascertained for the control group at a mean of 108 and 81 days after the first and second doses of anti-SARS-CoV-2 vaccine. The main difference between myocarditis patients and the comparator population was in terms of their history of previous SARS-CoV-2 infection (25 vs. 91%); however, anti-NCP (IgG, IgM) testing showed no difference between the two groups. There was no significant difference in the humoral immune response of myocarditis patients after SARS-CoV-2 immunization and those of sex- and age-matched controls (male patients, 22 ± 7 vs. 22 ± 6 years) (Table 5). In contrast, we found an increased T-cell response in myocarditis patients compared to controls (*P* < 0.01). We found that S1 IgG and IgA values negatively correlated with the time elapsed since the first vaccination (Supplementary Figure 1). Markers of the humoral immune response showed higher values after the mRNA vaccine than after the vector vaccine. At the same time, there was no difference regarding the cellular immune response between the two groups (Supplementary Table 1).

**Table 5:** Immune response in myocarditis patients after COVID-19 immunization vs. age-, sex- and COVID-19 immunization-matched controls.

	**Myocarditis patients after COVID-19 vaccination** **(*n =* 12)**	**Age- sex- and immunization-matched controls (*n* = 23)**	***P***
Age, years	22 ± 7	22 ± 6	0.924
Sex, male %	12 (100)	23 (100)	NA
Time from the first dose of vaccine to test, days	109 ± 57	108 ± 58	0.983
Time form the second dose of vaccine to test, days	86 ± 60	81 ± 55	0.907
COVID-19 vaccine			
- mRNA vaccine *n* (%)	8 (67%)	18 (78%)	0.814
- vector vaccine *n* (%)	4 (33%)	5 (22%)	
Test after the second dose of COVID-19 vaccine, yes (*n* %)	10 (83%)	18 (86%)	0.432
Previous SARS-CoV-2 infection, yes *n* (%)	3 (25%)	21 (91%)	<0.001
Time from previous SARS-CoV-2 infection, days	224 ± 66	284 ± 73	0.206
Anti-SARS-CoV-2 NCP-IgG (Ratio^*^) *Cutoff: > 1.1*	0.24 [0.13, 0.49]	0.32 [0.21, 1.23]	0.198
Anti-SARS-CoV-2 NCP-IgM (Ratio^*^) *Cutoff: > 1.1*	0.31 [0.24, 0.48]	0.33 [0.18, 0.66]	0.715
S1 Ig (U/ml) *Cutoff: ≥ 0.8 U/ml*	10265.5 [2,232, 38327.5]	9,167 [3948.5, 20,050]	0.881
SP1 IgG (RU/ml) *Cutoff: ≥ 11 RU/ml*	1155.5 [284, 1,656]	627 [283, 1537.5]	0.505
SP1 IgA (Ratio^*^) *Cutoff: ≥ 1.1*	11 [7, 11]	7 [6.5, 10]	0.095
Ag1 – S1 CD4+ (IU/ml) *Cutoff: ≥ 0.15*	1.3 [0.5, 4.5]	0.5 [0.2, 1.0]	0.002
Ag2 – S1 CD4+ CD8+ (IU/ml) *Cutoff: ≥ 0.15*	2.0 [1.0, 4.7]	0.6 [0.2, 1.2]	0.008
Ag3 – S1 CD4+ CD8+, whole genome CD8+ (IU/ml) *Cutoff: ≥ 0.15*	2.4 [1.0, 6.8]	0.8 [0.6, 1.5]	<0.001

Immune response to myocarditis after COVID-19 vaccination vs. age-, sex- and COVID-19 immunization-matched controls. Continuous variables showing a normal distribution are presented as the mean and standard deviations (± SD), and those showing a non-normal distribution are reported as medians and interquartile ranges [IQRs]. Comparisons between participant groups were conducted using independent samples t tests and Mann–Whitney U tests as appropriate.

* Ratio, extinction of the sample/extinction of calibrator; Ag, Antigen; CD, Cluster of differentiation; NA, Not applicable; NCP, Nucleocapsid protein; SARS-CoV-2, Severe acute respiratory syndrome coronavirus 2; SP1, Spike protein 1.

Notably, there was no difference in the immune response of myocarditis patients with or without predisposing factors (Supplementary Table 2).

**Figure 5:**
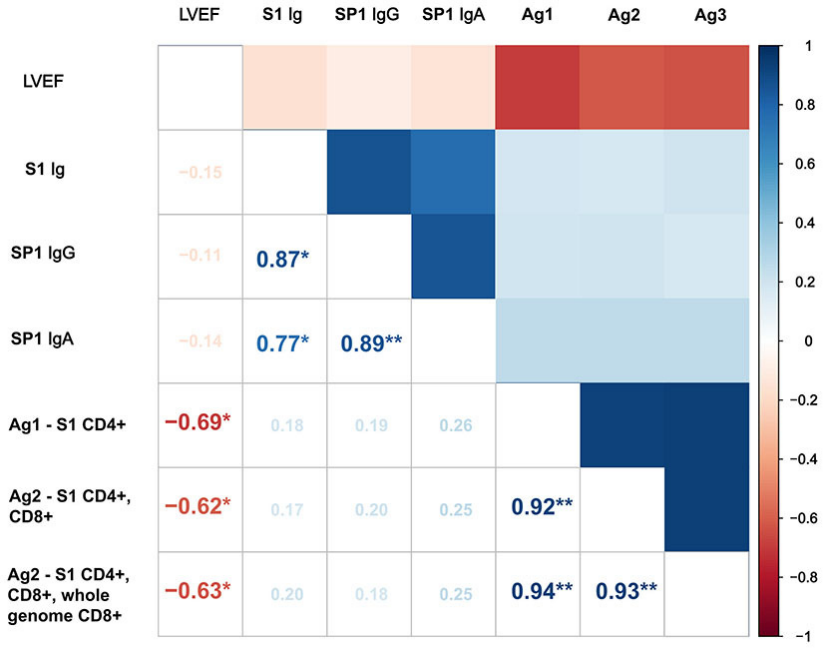
Correlation matrix showing the associations between SARS-CoV-2 immune response and LVEF. Positive correlations are shown in blue, and negative correlations are shown in red. Ag, Antigen; CD, Cluster of differentiation; SARS-CoV-2, Severe acute respiratory syndrome coronavirus 2; SP1, Spike protein 1; *p < 0.05; **p < 0.001.

Finally, there was no correlation between the humoral immune response (S Ig, SP1 IgG, SP1 IgA) and LVEF. In contrast, we found that the T-cell response parameters showed a negative correlation with the marker of systolic function (Figure 5).

## Discussion

### Summary of findings

The present data confirm and extend previous observations regarding the association of COVID-19 vaccination with myocarditis. This study of myocarditis patients after COVID-19 immunization confirmed by CMR makes the following contributions. First, in a cohort of acute myocarditis presenting a mean of 4 days after COVID-19 vaccination, we found that 75% had received mRNA vaccines and 25% vector vaccines. Second, on the follow-up visit, a mean of 112 days after the acute presentation, CMR abnormalities depicting myocardial injury, decreased, or completely disappeared. Third, there was no apparent difference regarding CMR metrics between myocarditis cases potentially associated with COVID-19 vaccination and myocarditis unrelated to COVID-19. Finally, we found an increased T-cell response among myocarditis patients after vaccination compared to matched controls.

### Comparison with existing literature

Our patients invariably presented with fever followed by chest pain and elevated troponin levels, typically 2–4 days after the second dose of the COVID-19 vaccine. This finding is consistent with previous reports (1, 12, 18). There was no evidence of ongoing SARS-CoV-2 infection or other viral infection in any of the participants. While most of our patients presented after the mRNA vaccine, similar to what studies from the US and Israel found (1, 13), 25% of all cases presented after receiving the Sputnik V vaccine. In Hungary, ~40% of the population between the ages of 16 and 35 received a vector anti-SARS-CoV-2 vaccine (19), suggesting that myocarditis after COVID-19 vaccine might be less skewed toward mRNA vaccines than previously reported (20). Notably, at the time of our study, only the Pfizer-BioNTech vaccine was authorized to immunize the adolescent population (*n* = 5 in our cohort), who seem to be more prone to this adverse effect (4). This might limit meaningful comparison of the risk of myocarditis associated with different COVID-19 vaccines. Interestingly, a study based on the Vaccine Adverse Events Reporting System (VAERS) already cautioned against using mRNA vaccines among those with a higher risk for myocarditis and encourages vector vaccines as a safer alternative (20). However, a passive reporting system such as VAERS is prone to over- or underreporting based on the knowledge and attention of the reporters (5). Therefore, it should be used as a hypothesis-generating or event detection system (5, 21). Moreover, participants in our study received Gam-COVID-Vac (two doses required) as opposed to the Janssen vaccine (one dose required), which is approved by the Food and Drug Administration for use in the US and is therefore reported in the VAERS.

There are several aspects of the history of our patients that are worth noting. Twenty-five percent of our patients reported immune-mediated diseases. Furthermore, two individuals reported prior acute myocarditis, and one experienced recurrent myocarditis 3 months after vaccination. In the latter case, acute myocarditis was linked to acute gastrointestinal infection; thus, it seems unlikely that this event was associated with vaccination. These findings might suggest a predisposing immune system response, as described previously in the etiology of acute myocarditis unrelated to vaccination (22). We did not find a statistically significant difference between the immune response of participants with predisposing factors and that of those without predisposing factors; however, the limited number of patients in each group precludes meaningful conclusions.

The male predominance of myocarditis after vaccination and myocarditis unrelated to vaccination has been previously described, and the cause is still unknown (23). One leading hypothesis is based on sex hormone disparities. It has been proven that there are differences in sex hormone receptor expression on both immune cells and cardiac tissues (24). The highest free testosterone levels have been described in males aged 12–24 years (25). Moreover, testosterone has a role in interleukin-10 upregulation and interferon-gamma downregulation. However, the direct relationship between testosterone levels and myocarditis has not been conclusively proven. Finally, experimental data demonstrate that Y chromosome-associated genetic factors are also responsible for the higher prevalence of myocarditis among males (26). Vigorous sports activity can trigger the onset of acute myocarditis and should be avoided during ongoing infection (22, 27); this might also be applicable after immunization, especially among young males. Five individuals reported possible acute triggers in our cohort: vigorous physical activity (*n* = 4) and heavy alcohol consumption (*n* = 1) immediately after immunization. In summary, our current findings suggest that the combined effect of genetic predisposition, hormonal factors and acute triggers may contribute to the pathomechanism of myocarditis after COVID-19 vaccination.

Several case reports have provided a visual account of myocarditis after COVID-19 immunization using CMR imaging (28–31), and this is the first study to show the improvement of myocardial injury. Moreover, for context, we provided a control group of myocarditis unrelated to the COVID-19 vaccine or SARS-CoV-2 infection. In our study, the most frequent localization of LGE was the lateral wall of the left ventricle in both myocarditis patients after COVID-19 vaccination and patients with myocarditis unrelated to COVID-19 infection or vaccination. This suggests that based on the CMR image, it is impossible to distinguish myocarditis cases post-vaccination from viral myocarditis. Our finding is in line with the recent report from Fronza et al. (32). CMR is a crucial diagnostic tool for myocardial injury. However, clarifying the disease etiology requires a holistic approach, taking into account the patient's history, symptoms and potential predisposing factors.

It has been shown, that acute myocarditis can heal or completely resolve over time (33), and our results support the notion that this is also true for cases potentially linked to the COVID-19 vaccine. We found that T2 mapping returned to the normal range on follow-up for all patients. Moreover, T1 mapping, ECV, and LGE decreased. Data suggest that LGE on the acute CMR scan is not equal to irreversible myocardial damage but the result of myocardial inflammation that can decrease over time and suggests a better prognosis over more extended follow-up periods. Additionally, none of the participants had extensive (>20%) LGE during follow-up, which is also considered a better prognostic marker (27). We found a slight improvement in LVEF during follow-up. Whilst the betterment of GLS values were not significant in our study, as expected based on the literature (34), the overall trend of GLS also suggested a marginal improvement over time when looking at individual data points.

In addition to the production of SARS-CoV-2-specific antibodies, COVID infection also leads to the generation of specific CD4+ and CD8+ cells (35). Increasing evidence supports the essential role of the T-cell-mediated response to SARS-CoV-2 infection; the COVID-specific T-cell response is associated with less severe disease (36, 37). Thereafter, to obtain a comprehensive view regarding the COVID-specific adaptive immune response, it is essential to measure specific antibodies and CD4+ and CD8+ cells from the same individual. Our current data indicate a substantially accelerated COVID-specific T-cell-mediated immune response in the myocarditis group compared to the age-, sex-, and vaccination status-adjusted control population. It is noteworthy that a larger proportion of controls than myocarditis patients had previously had COVID infections.

The rapid onset of symptoms after vaccination is an intriguing phenomenon and might be connected with immune response-mediated pathomechanisms. Reports all over the globe agree that myocarditis starts ~2–4 days after vaccination. Although data regarding long-term immunity are scarce, it seems that a T-cell response is sustained for several months after infection and appears to be more prolonged than the antibody response. It has also been suggested that the T-cell response to different COVID-19 vaccines differs among age groups (17).

While we believe that acute myocarditis after COVID-19 vaccination is an important cardiovascular adverse effect that may occur after both mRNA and vector vaccines, this should not overshadow the ample evidence that clearly supports the effectiveness of vaccines (38, 39). The question also arose if young patients with COVID-19 are more likely to develop acute myocarditis or other adverse events than individuals after SARS-CoV-2 immunization. The most serious of which is the multisystem inflammatory syndrome in children (MIS-C). Recent evidence from France suggests that COVID-19 vaccination is associated with lower MIS-C incidence among adolescents (40). Moreover, in a new report by Zambrano et al. critically ill MIS-C patients requiring life support, all were unvaccinated, reinforcing the COVID-19 vaccination recommendation for eligible children (41). Therefore, there is an urgent need for an international consensus recommendation regarding an immunization protocol for those who experienced acute myocarditis after their COVID-19 vaccine.

## Limitations

The main limitation of our study is the small sample size, which is mainly due to the rare occurrence of myocarditis after COVID-19 vaccination. Although we contacted all Hungarian centers reporting CMR, we could not avoid referral bias to CMR by clinicians. Mapping sequences were available in three institutes out of four. Similarly to other reports of myocarditis after COVID-19 vaccination, we report myocarditis cases of young, male patients. This prevents generalizability of our results to the female or older male population. In the institute where the parametric T2 mapping sequence was not available, oedema was characterized by T2-weighted black blood images alone. Mapping sequences were compared only among those participants who were scanned at the Semmelweis University Heart and Vascular Center (using a Siemens Magnetom Aera 1.5 T scanner) to avoid inter scanner variability. Importantly, our myocarditis control group's history was provided by the referring physician. The control group for the immunological studies did not undergo CMR examination.

## Conclusions

In this cohort of myocarditis patients after COVID-19 immunization confirmed by CMR, we found that acute myocarditis can occur after mRNA and vector vaccines, predominantly in individuals with predisposing factors. Upon mid-term follow-up, myocarditis showed improvements in CMR markers, including the LVEF and tissue-specific alterations. The T-cell response was more prominent among myocarditis patients after COVID-19 vaccination than matched controls.

## Data availability statement

The original contributions presented in the study are included in the article/Supplementary material, further inquiries can be directed to the corresponding authors.

## Ethics statement

The studies involving human participants were reviewed and approved by National Public Health Center of Hungary. Written informed consent to participate in this study was provided by the participants' legal guardian/next of kin. Written informed consent was obtained from the individual(s) for the publication of any potentially identifiable images or data included in this article.

## Author contributions

HV, LS, GN, BM, RK, and DB contributed to the conception and design of the study. HV, LS, AT, VJ, ZD, GB, ES, ZU, ZS, and GGN contributed to the data acquisition and curation. LS performed the statistical analysis and wrote the first draft of the manuscript. HV refined the manuscript. GN, GGN, ZU, and ZS wrote sections of the manuscript. All authors contributed to manuscript revision, read, and approved the submitted version.

## Funding

This study was financed by the Research Excellence Programme of the Ministry for Innovation and Technology in Hungary within the framework of the Bioimaging Thematic Programme of Semmelweis University and by the Ministry of Innovation and Technology NRDI Office within the framework of the Artificial Intelligence National Laboratory Program. This project was supported by a grant from the National Research, Development and Innovation Office (NKFIH) of Hungary (K135076) to BM. This project was supported by a grant from the National Research, Development and Innovation Office (NKFIH) of Hungary (2020-1.1.6-JÖVO-2021-00013) and the Development of scientific workshops of medical, health sciences and pharmaceutical educations project. Project identification number: EFOP-3.6.3-VEKOP-16-2017-00009. LS received funding from the European Association of Cardiovascular Imaging (EACVI Research Grant App000076437). Project no. RRF-2.3.1-21-2022-00003 has been implemented with the support provided by the European Union.

## Conflict of interest

The authors declare that the research was conducted in the absence of any commercial or financial relationships that could be construed as a potential conflict of interest.

## Publisher's note

All claims expressed in this article are solely those of the authors and do not necessarily represent those of their affiliated organizations, or those of the publisher, the editors and the reviewers. Any product that may be evaluated in this article, or claim that may be made by its manufacturer, is not guaranteed or endorsed by the publisher.

## References

[1] Montgomery J Ryan M Engler R Hoffman D Mcclenathan B Collins L. Myocarditis following immunization with mRNA COVID-19 vaccines in members of the US Military. JAMA Cardiol. (2021) 6:1202–6. 10.1001/jamacardio.2021.2833 34185045 PMC8243257

[2] Dionne A Sperotto F Chamberlain S Baker AL Powell AJ Prakash A. Association of myocarditis with BNT162b2 messenger RNA COVID-19 vaccine in a case series of children supplemental content. JAMA Cardiol. (2021) 6:1446–50. 10.1001/jamacardio.2021.3471 34374740 PMC8356143

[3] Witberg G Barda N Hoss S Richter I Wiessman M Aviv Y. Myocarditis after COVID-19 vaccination in a large health care organization. N Engl J Med. (2021) 385:2132–41. 10.1056/NEJMoa2110737 34614329 PMC8531986

[4] Bozkurt B Kamat I Hotez PJ. Myocarditis with COVID-19 mRNA vaccines. Circulation. (2021) 2019:471–84. 10.1161/CIRCULATIONAHA.121.056135 34281357 PMC8340726

[5] Shimabukuro TT Nguyen M Martin D DeStefano F. Safety monitoring in the Vaccine Adverse Event Reporting System (VAERS). Vaccine. (2015) 33:4398–405. 10.1016/j.vaccine.2015.07.035 26209838 PMC4632204

[6] Kramer CM Barkhausen J Bucciarelli-Ducci C Flamm SD Kim RJ Nagel E. Standardized cardiovascular magnetic resonance imaging (CMR) protocols: 2020 update. J Cardiovasc Magn Reson. (2020) 22:17. 10.1186/s12968-020-00607-1 32089132 PMC7038611

[7] Ferreira VM Schulz-Menger J Holmvang G Kramer CM Carbone I Sechtem U. Cardiovascular magnetic resonance in nonischemic myocardial inflammation: expert recommendations. J Am Coll Cardiol. (2018) 72:3158–76. 10.1016/j.jacc.2018.09.072 30545455

[8] Leiner T Bogaert J Friedrich MG Mohiaddin R Muthurangu V Myerson S. SCMR position paper (2020) on clinical indications for cardiovascular magnetic resonance. J Cardiovasc Magn Reson. (2020) 22:76. 10.1186/s12968-020-00682-4 33161900 PMC7649060

[9] Ackerman M Atkins DL Triedman JK. Sudden cardiac death in the young. Circulation. (2016) 133:1006–26. 10.1161/CIRCULATIONAHA.115.020254 26951821 PMC5033115

[10] George B Seals S Aban I. Survival analysis and regression models. J Nucl Cardiol. (2014) 21:686–94. 10.1007/s12350-014-9908-2 24810431 PMC4111957

[11] Rosseel Y. Lavaan: an R package for structural equation modeling. J Stat Softw. (2012) 48:1–36. 10.18637/jss.v048.i0225601849

[12] Kim HW Jenista ER Wendell DC Azevedo CF Campbell MJ Darty SN. Patients with acute myocarditis following mRNA COVID-19 vaccination. JAMA Cardiol. (2021) 6:1196–201. 10.1001/jamacardio.2021.2828 34185046 PMC8243258

[13] Mevorach D Anis E Cedar N Bromberg M Haas EJ Nadir E. Myocarditis after BNT162b2 mRNA vaccine against COVID-19 in Israel. N Engl J Med. (2021) 385:2140–9. 10.1056/NEJMoa2109730 34614328 PMC8531987

[14] Marrama D Mahita J Sette A Peters B. Lack of evidence of significant homology of SARS-CoV-2 spike sequences to myocarditis-associated antigens. eBioMedicine. (2022) 75:103807. 10.1016/j.ebiom.2021.103807 34998242 PMC8733122

[15] Schulz-Menger J Bluemke DA Bremerich J Flamm SD Fogel MA Friedrich MG. Standardized image interpretation and post-processing in cardiovascular magnetic resonance - 2020 update: Society for Cardiovascular Magnetic Resonance (SCMR): Board of Trustees Task Force on Standardized Post-Processing. J Cardiovasc Magn Reson. (2020) 22:19. 10.1186/s12968-020-00610-6 32160925 PMC7066763

[16] Elslande JV Houben E Depypere M Brackenier A Desmet S André E. Diagnostic performance of seven rapid IgG/IgM antibody tests and the Euroimmun IgA/IgG ELISA in COVID-19 patients. Clin Microbiol Infect. (2020) 26:1082–7. 10.1016/j.cmi.2020.05.023 32473953 PMC7255746

[17] Jaganathan S Stieber F Rao SN Nikolayevskyy V Manissero D Allen N. Preliminary evaluation of QuantiFERON SARS-CoV-2 and QIAreach Anti-SARS-CoV-2 total test in recently vaccinated individuals. Infect Dis Ther. (2021) 10:2765–76. 10.1007/s40121-021-00521-8 34435336 PMC8386336

[18] Diaz GA Parsons GT Gering SK Meier AR Hutchinson IV Robicsek A. Myocarditis and Pericarditis after vaccination for COVID-19. JAMA. (2021) 326:1210–2. 10.1001/jama.2021.13443 34347001 PMC8340007

[19] Vokó Z Kiss Z Surján G Surján O Barcza Z Pályi B. Nationwide effectiveness of five SARS-CoV-2 vaccines in Hungary—the HUN-VE study. Clin Microbiol Infect. (2021) 28:398–404. 10.1016/j.cmi.2021.11.011 34838783 PMC8612758

[20] Li M Yuan J Lv G Brown J Jiang X Lu ZK. Myocarditis and Pericarditis following COVID-19 vaccination : inequalities in age and vaccine types. J Pers Med. (2021) 11:1106. 10.3390/jpm11111106 34834458 PMC8624452

[21] Mei R Raschi E Forcesi E Diemberger I De Ponti F Poluzzi E. Myocarditis and pericarditis after immunization: gaining insights through the Vaccine Adverse Event Reporting System. Int J Cardiol. (2018) 273:183–6. 10.1016/j.ijcard.2018.09.054 30236504

[22] Caforio ALP Pankuweit S Arbustini E Basso C Gimeno-Blanes J Felix SB. Current state of knowledge on aetiology, diagnosis, management, and therapy of myocarditis: a position statement of the European Society of Cardiology Working Group on Myocardial and Pericardial Diseases. Eur Heart J. (2013) 34:2636–48. 10.1093/eurheartj/eht210 23824828

[23] Gargano J Wallace M Hadler S Langley G JR S Oster M. Use of mRNA COVID-19 vaccine after reports of myocarditis among vaccine recipients: update from the advisory committee on immunization practices — United States, June (2021). Morb Mortal Wkly Rep. (2021) 70:977–82. 10.15585/mmwr.mm7027e2 34237049 PMC8312754

[24] Di Florio DN Sin J Coronado MJ Atwal PS Fairweather DL. Sex differences in inflammation, redox biology, mitochondria and autoimmunity. Redox Biol. (2020) 31:101482. 10.1016/j.redox.2020.101482 32197947 PMC7212489

[25] Senefeld J Lambelet Coleman D Johnson P Carter R Clayburn A Joyner M. Divergence in timing and magnitude of testosterone levels between male and female youths. JAMA. (2020) 382:2368–71. 10.1001/jama.2020.5655 32633795 PMC7341166

[26] Case LK Wall EH Dragon JA Saligrama N Krementsov DN Moussawi M. The y chromosome as a regulatory element shaping immune cell transcriptomes and susceptibility to autoimmune disease. Genome Res. (2013) 23:1474–85. 10.1101/gr.156703.113 23800453 PMC3759723

[27] Task A Members F Pelliccia A France JC Drezner JA States U. 2020 ESC Guidelines on sports cardiology and exercise in patients with cardiovascular disease. Eur Heart J. (2020) 1–80. 32860412 10.1093/eurheartj/ehaa605

[28] Singh B Kaur P Cedeno L Brahimi T Patel P Virk H. COVID-19 mRNA vaccine and myocarditis. Eur J Case Rep Intern Med. (2021) 8:002681. 10.12890/2021_002681 34268277 PMC8276934

[29] D'Angelo T Cattafi A Carerj ML Booz C Ascenti G Cicero G. Myocarditis after SARS-CoV-2 vaccination: a vaccine-induced reaction? Can J Cardiol. (2021). 37:1665–7. 10.1016/j.cjca.2021.05.010 34118375 PMC8187737

[30] Shaw KE Cavalcante JL Han BK Gössl M. Possible association between COVID-19 vaccine and myocarditis: clinical and CMR findings. JACC Cardiovasc Imaging. (2021) 14:1856–61. 10.1016/j.jcmg.2021.06.002 34246586 PMC8245050

[31] Mansour J Short RG Bhalla S Woodard PK Verma A Robinson X. Acute myocarditis after a second dose of the mRNA COVID-19 vaccine: a report of two cases. Clin Imaging. (2021) 78:247–9. 10.1016/j.clinimag.2021.06.019 34166884 PMC8216670

[32] Fronza M Thavendiranathan P Chan V Karur GR Udell JA Wald RM. Myocardial injury pattern at MRI in COVID-19 vaccine–associated Myocarditis. Radiology. (2022) 212559. 10.1148/radiol.212559 35166587 PMC8856022

[33] Aquaro GD Ghebru Habtemicael Y Camastra G Monti L Dellegrottaglie S Moro C. Prognostic value of repeating cardiac magnetic resonance in patients with acute Myocarditis. J Am Coll Cardiol. (2019) 74:2439–48. 10.1016/j.jacc.2019.08.1061 31727281

[34] Porcari A Merlo M Baggio C Gagno G Cittar M Barbati G. Global longitudinal strain by CMR improves prognostic stratification in acute myocarditis presenting with normal LVEF. Eur J Clin Invest. (2022) 29:zwac056.066. 10.1093/eurjpc/zwac056.066 35598175

[35] Sette A Crotty S. Adaptive immunity to SARS-CoV-2 and COVID-19. Cell. (2021) 184:861–80. 10.1016/j.cell.2021.01.007 33497610 PMC7803150

[36] Liao M Liu Y Yuan J Wen Y Xu G Zhao J. Single-cell landscape of bronchoalveolar immune cells in patients with COVID-19. Nat Med. (2020) 26:842–4. 10.1038/s41591-020-0901-9 32398875

[37] Rydyznski Moderbacher C Ramirez SI Dan JM Grifoni A Hastie KM Weiskopf D. Antigen-specific adaptive immunity to SARS-CoV-2 in acute COVID-19 and associations with age and disease severity. Cell. (2020) 183:996–1012.e19. 10.1016/j.cell.2020.09.038 33010815 PMC7494270

[38] Olson SM Newhams MM Halasa NB Price AM Boom JA Sahni LC. Effectiveness of BNT162b2 vaccine against critical COVID-19 in adolescents. N Engl J Med. (2022) 386:713–23. 10.1056/NEJMoa2117995 35021004 PMC8781318

[39] Polack FP Thomas SJ Kitchin N Absalon J Gurtman A Lockhart S. Safety and efficacy of the BNT162b2 mRNA COVID-19 vaccine. N Engl J Med. (2020) 383:2603–15. 10.1056/NEJMoa2034577 33301246 PMC7745181

[40] Levy M Recher M Hubert H Javouhey E Fléchelles O Leteurtre S. Multisystem inflammatory syndrome in children by COVID-19 vaccination status of adolescents in France. JAMA - J Am Med Assoc. (2021). 34928295 10.1001/jama.2021.23262 PMC8689418

[41] Zambrano LD Newhams MM Olson SM Halasa NB Price AM. Effectiveness of BNT162b2 (Pfizer-BioNTech) mRNA vaccination against multisystem inflammatory syndrome in children among persons aged 12 – 18 years — United States, July – December 2021. MMWR Morb Mortal Wkly Rep. (2022) 71:52–8. 10.15585/mmwr.mm7102e1 35025852 PMC8757620

