# Supplementary material for: Immunological response and temporal associations in myocarditis after COVID-19 vaccination using cardiac magnetic resonance imaging: An amplified T-cell response at the heart of it?
Source: Front Cardiovasc Med. 2022 Sep 15;9:961031. doi: 10.3389/fcvm.2022.961031 (PMC9520979; doi:10.3389/fcvm.2022.961031)
Supplement: Supplementary file 1 [file Table_1.DOCX]

Supplementary Table 1 Immune response to mRNA vs vector vaccine

|  | mRNA vaccine  (n=26) | Vector vaccine  (n=9) | P |
| --- | --- | --- | --- |
| Age, years | 22 ± 6 | 22 ± 4 | NS |
| Time from the first vaccine to test | 111 ± 59 | 102 ± 53 | NS |
| S1 Ig (U/ml)  *Cutoff: ≥ 0.8 U/ml* | 13252 [5848, 27727] | 3127 [428.5, 8785] | 0.015 |
| SP1 IgG (RU/ml)  *Cutoff: ≥ 11 RU/ml* | 1087 [353, 2089.5] | 284 [68, 507.5] | 0.006 |
| SP1 IgA (Ratio*)  *Cutoff: ≥ 1.1* | 8.6 [6.9, 11.2] | 5.6 [1.9, 8.1] | 0.017 |
| Ag1 – S1 CD4+ (IU/ml)  *Cutoff: ≥ 0.15* | 0.8 [0.4, 1.4] | 0.3 [0.1, 1.4] | NS |
| Ag2 – S1 CD4+ CD8+ (IU/ml)  *Cutoff: ≥ 0.15* | 1 [0.6, 1.7] | 0.6 [0.2, 1.8] | NS |
| Ag3 – S1 CD4+ CD8+, whole genome CD8+ (IU/ml)  *Cutoff: ≥ 0.15* | 1.2 [0.8, 2.1] | 0.7 [0.4, 2.3] | NS |

Supplementary Table 2: Myocarditis after COVID-19 vaccination. Comparison between patients with predisposing factors and without.

|  | Myocarditis after COVID-19 vaccination with predisposing factors  (n=5*) | Myocarditis after COVID-19 vaccination without predisposing factors  (n=7) | P |
| --- | --- | --- | --- |
| S1 Ig (U/ml)  *Cutoff: ≥ 0.8 U/ml* | 7279 [3114, 41911] | 13252 [1954, 30768] | NS |
| SP1 IgG (RU/ml)  *Cutoff: ≥ 11 RU/ml* | 1656 [477, 2428] | 771 [309, 1603] | NS |
| SP1 IgA (Ratio*)  *Cutoff: ≥ 1.1* | 10 [5.4, 11.3] | 11.1 [8.3, 11.3] | NS |
| Ag1 – S1 CD4+ (IU/ml)  *Cutoff: ≥ 0.15* | 2.6 [0.8, 6.8.8] | 1 [0.4, 2.2] | NS |
| Ag2 – S1 CD4+ CD8+ (IU/ml)  *Cutoff: ≥ 0.15* | 4.6 [1.8, 5.7] | 1.7 [0.9, 3.1] | NS |
| Ag3 – S1 CD4+ CD8+, whole genome CD8+ (IU/ml)  *Cutoff: ≥ 0.15* | 6.6 [1.9, 7.4] | 1.8 [0.9, 3.2] | NS |

* Crohn’s disease (n=1), psoriasis (n=1), allergies (n=1), previous myocarditis (n=1), asthma and previous myocarditis (n=1)
