# Supplementary figures and images for: Immunological response and temporal associations in myocarditis after COVID-19 vaccination using cardiac magnetic resonance imaging: An amplified T-cell response at the heart of it?
Source: Front Cardiovasc Med. 2022 Sep 15;9:961031. doi: 10.3389/fcvm.2022.961031 (PMC9520979; doi:10.3389/fcvm.2022.961031)

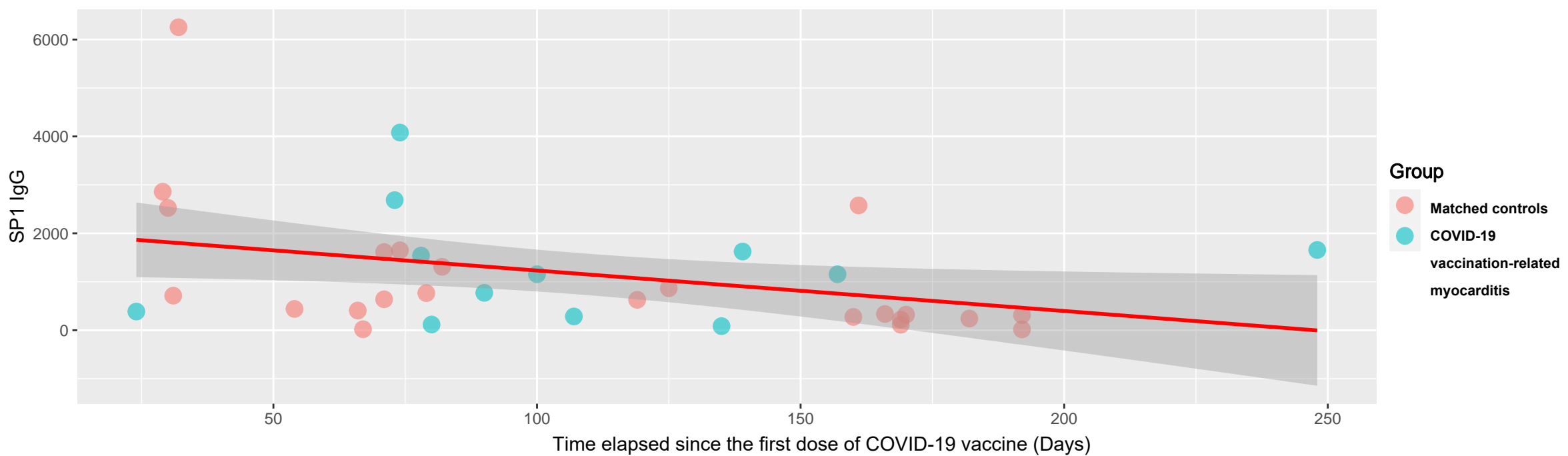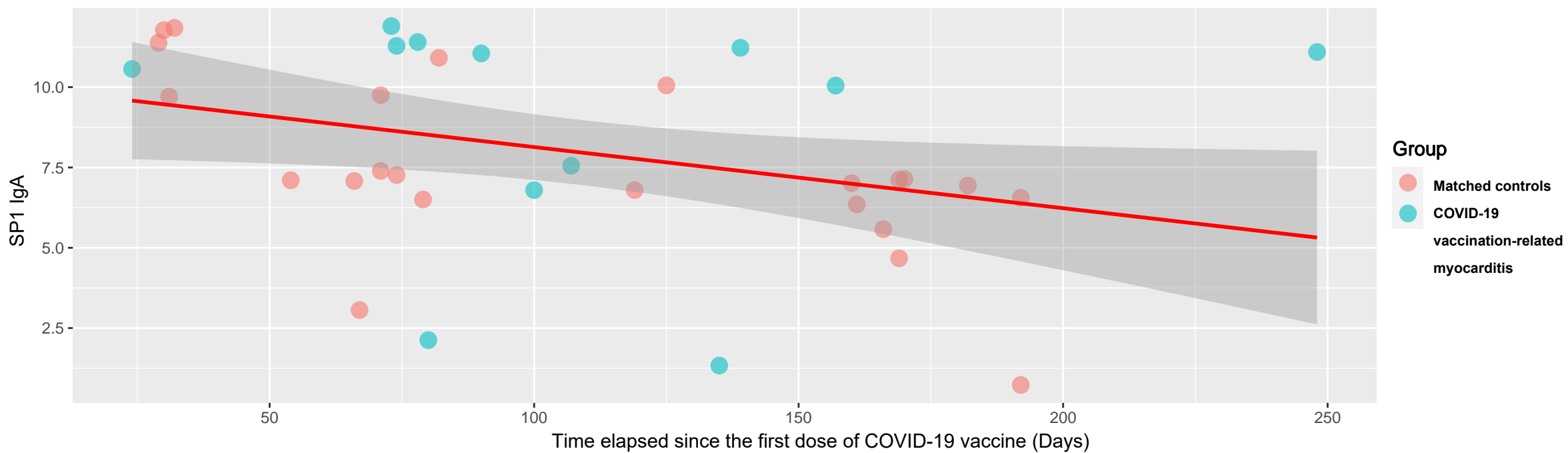

Supplement: Supplementary file 2 [file Data_Sheet_1.PDF]
