# Supplementary material for: Immunological response and temporal associations in myocarditis after COVID-19 vaccination using cardiac magnetic resonance imaging: An amplified T-cell response at the heart of it?
Source: Front Cardiovasc Med. 2022 Sep 15;9:961031. doi: 10.3389/fcvm.2022.961031 (PMC9520979; doi:10.3389/fcvm.2022.961031)
Supplement: Supplementary file 3 [file Data_Sheet_2.PDF]

Percentage and colour code shows the frequency with which a given AHA segment was affected on the acute CMR scan

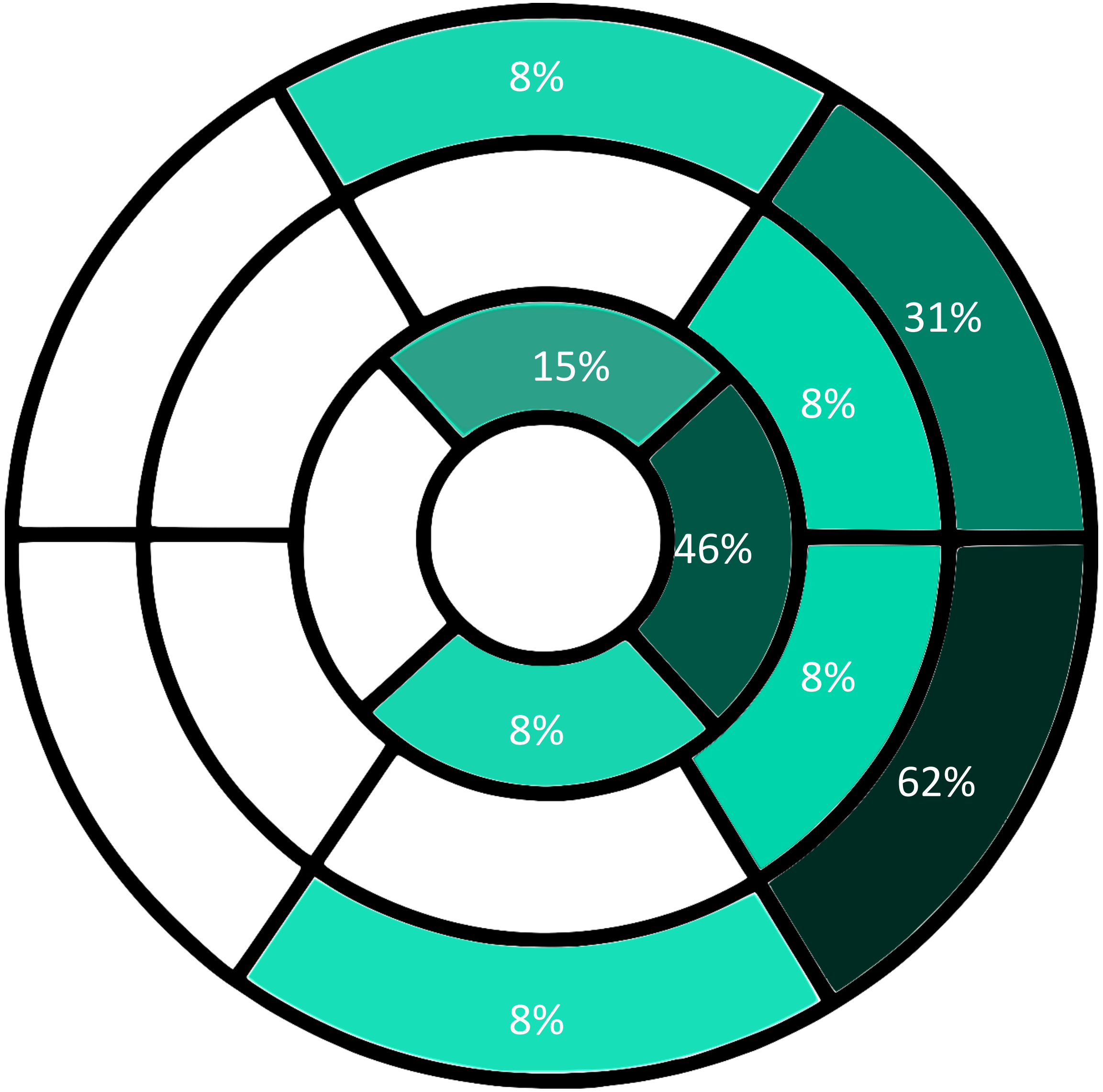

Myocarditis after COVID-19 vaccination

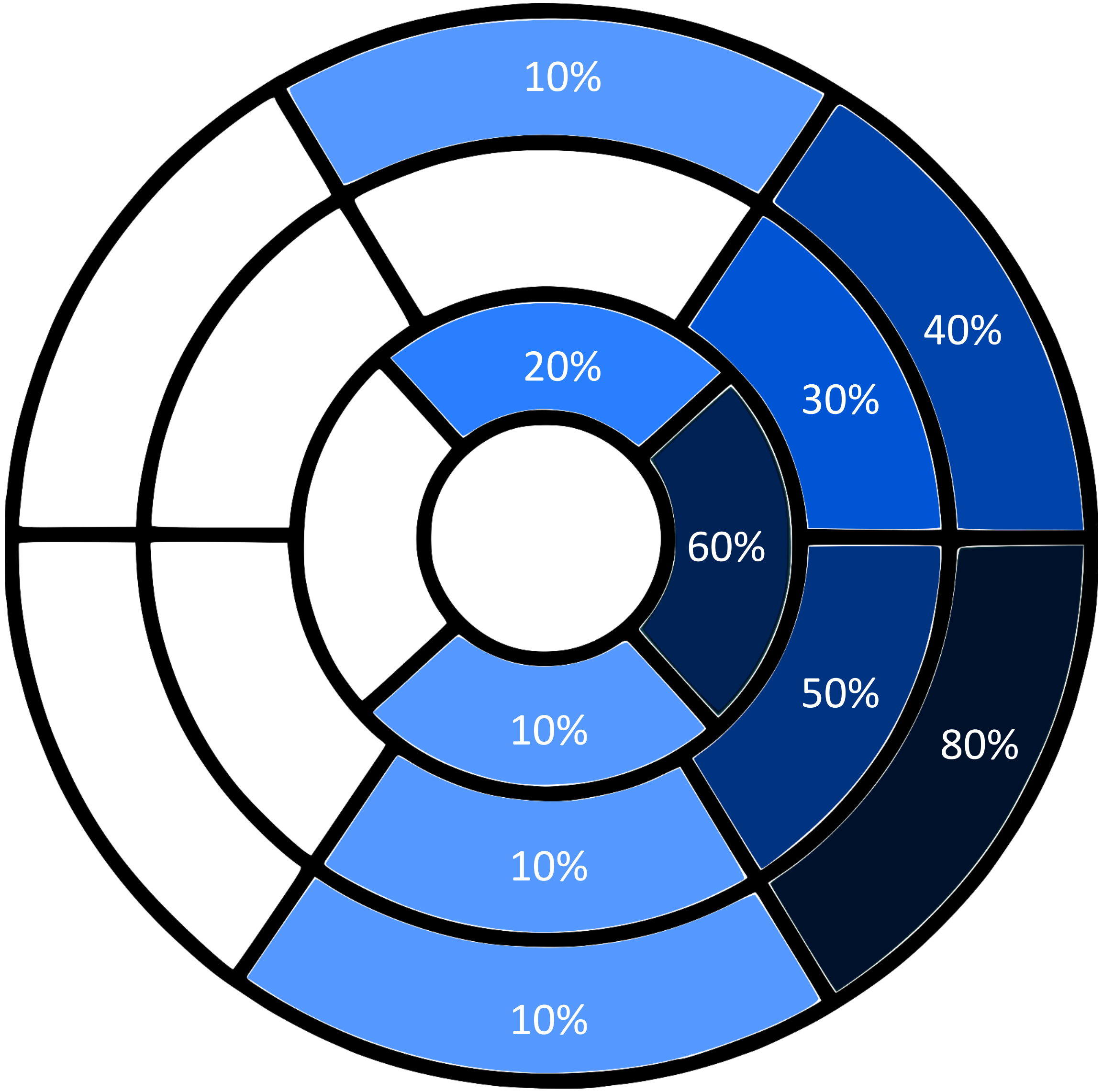

Myocarditis in patients with no COVID-19 infection or vaccination
